# Design and Electrochemical Study of Platinum-Based Nanomaterials for Sensitive Detection of Nitric Oxide in Biomedical Applications

**DOI:** 10.3390/nano6110211

**Published:** 2016-11-14

**Authors:** Maduraiveeran Govindhan, Zhonggang Liu, Aicheng Chen

**Affiliations:** Department of Chemistry, Lakehead University, 955 Oliver Road, Thunder Bay, ON P7B 5E1, Canada; mgovindh@lakeheadu.ca (M.G.); zliu18@lakeheadu.ca (Z.L.)

**Keywords:** platinum, tungsten, nanomaterials, modified electrodes, electrocatalysis, electrochemical sensors, nitric oxide, biomedical applications

## Abstract

The extensive physiological and regulatory roles of nitric oxide (NO) have spurred the development of NO sensors, which are of critical importance in neuroscience and various medical applications. The development of electrochemical NO sensors is of significant importance, and has garnered a tremendous amount of attention due to their high sensitivity and selectivity, rapid response, low cost, miniaturization, and the possibility of real-time monitoring. Nanostructured platinum (Pt)-based materials have attracted considerable interest regarding their use in the design of electrochemical sensors for the detection of NO, due to their unique properties and the potential for new and innovative applications. This review focuses primarily on advances and insights into the utilization of nanostructured Pt-based electrode materials, such as nanoporous Pt, Pt and PtAu nanoparticles, PtAu nanoparticle/reduced graphene oxide (rGO), and PtW nanoparticle/rGO-ionic liquid (IL) nanocomposites, for the detection of NO. The design, fabrication, characterization, and integration of electrochemical NO sensing performance, selectivity, and durability are addressed. The attractive electrochemical properties of Pt-based nanomaterials have great potential for increasing the competitiveness of these new sensors and open up new opportunities in the creation of novel NO-sensing technologies for biological and medical applications.

## 1. Introduction

The sensing of nitric oxide (NO) molecules has fundamental importance in the elucidation of human cell functionality, pathology, and toxicity, as well as practical significance in the development of potential platforms for medical and environmental applications [1,2,3]. Due to its distinctive critical properties, but also its potential toxicity, NO has been cast as both hero and villain in the chemical and biological scientific world [4,5,6,7]. In particular, NO has been found to be released by various cells in mammalian systems, and plays a vital role in many biological processes, including the regulation of cell function in the nervous, vascular, and immune systems, neurotransmission, vasodilation, blood pressure, etc. [8,9,10]. Further, it is involved in a number of conditions such as cardiovascular disease, hypertension, symptoms of vaginitis, and cancer. In cancer, NO may act as either a tumor propagator or suppressor, which is primarily contingent on its concentration and lifetime within an organism [11,12,13]. The in vivo sensing of NO remains a constant challenge because of the trace level concentrations (~nanomolar) that are released from cells, high reactivity, and a short half-time (<10 s) via its rapid reaction with oxygen, thiols, free radicals, and hemes [14,15,16]. Therefore, the development of a robust and efficient NO sensor platform for practical medical applications remains a formidable task, as the sensor platform must be highly selective toward NO over interfering compounds, which is often challenging in biological systems.

Spectroscopic and electrochemical strategies are prominently employed analytical methods for the detection and determination of NO. Most spectroscopic measurements for NO detection comprise either the indirect measurement of byproducts generated by reactions between NO and other chemical species, or direct measurements of formed adducts between NO and metal complexes, fluorescent dyes, or spin traps with high sensitivity and selectivity [10]. However, the in vivo sensing of NO encounters significant challenges in the acquisition of spectroscopic measurements, due to the complex instrumentation involved and difficulties with miniaturization. Alternatively, electrochemical methods provide a platform for the direct measurement of NO with high analytical performance. Their positive attributes include high sensitivity and selectivity, rapid and stable response, easy use, low cost, easy miniaturization, and facilitating the measurement of real-time NO concentrations in biological samples, which have been employed over a wide range of sensors for clinical use or in biomedicine [8,17,18,19,20].

Nanostructured materials offer distinct advantages for numerous applications toward the development of a new generation of sensitive chemical and biological sensing platforms. In particular, the capacity for the identification of specific cell species or anatomical sites within the human body may bode very well for the use of nanobiosensors in medical diagnostics. There is great interest in the development of nanomaterial-based NO sensors, to enhance their surface-to-volume ratios, efficiently boost electron transfer, and establish stable responses [19,21,22,23]. It is recognized that NO molecules may easily adsorb onto the surfaces of metallic nanoparticles, and undergo electrochemical oxidation more easily than that with larger particles. Various functional nanomaterials, including metallic nanoparticles, have been investigated for the electrochemical sensing of NO, and exhibited improved performance [24,25,26].

Nanostructured Pt-based materials have garnered increasing attention over the last decade, in the areas of electrocatalysis and sensors, through the provision of high surface area to volume ratios, as well as the capacity to be tailored to encourage particular reaction pathways [27,28,29,30,31,32,33]. Morphologically dependent interatomic bond distances, melting points, chemical reactivity, as well as optical and electronic properties can have profound influences on the functionality of Pt nanomaterials. The chemical composition, surface condition, crystal structure quality, crystallographic axis orientation, etc. are acute parameters of Pt nanomaterials that cumulatively influence electron transport mechanisms [34,35,36]. The use of Pt-based nanocomposites in sensors may serve as one effective strategy toward the augmentation of their electronic, chemical, and electrochemical properties [17,36,37,38,39]. Platinum based nanocomposites such as bimetallic Pt-based alloy nanoparticles, Pt nanoparticles dispersed on various substrates such as metal oxides, carbon nanotubes (CNTs), reduced graphene oxide (rGO), and rGO/ionic liquid (IL) composites, may further enhance the oxidation of NO molecules due to their synergistic electronic effects [23,24,40,41,42,43]. Platinum based nanomaterials as sensing electrode materials may be added to the surfaces of electrodes or modified Pt electrodes through numerous strategies, including physical adsorption, chemical covalent bonding, electrochemical reduction, electrochemical deposition, electrochemical polymerization with redox polymers, etc. [20,34].

Platinum nanomaterial modified electrodes may have the ability to selectively adsorb and sense NO molecules. They may easily make possible the development of electrochemical sensor platforms with unique functionalities and processes that are activated on exposure to NO, as well as the capacity for enhanced mobility and the real time monitoring of other biomolecules in biomedical applications [19,23,44,45,46]. The main focus of this review is to provide insights into electrochemical NO sensors based on Pt nanomaterials. It will endeavor to describe the utility of Pt nanomaterials in the design of high-performance electrochemical sensor platforms for the detection of NO, which presently assist in the detection of various disease states, and facilitate the real-monitoring of biological processes. We are hopeful that the advances and concepts articulated in this review will inspire new discoveries in this area for the benefit of medical industries worldwide.

## 2. Nanoporous Platinum

Pt nanomaterial-based electrochemical sensor platforms are under widespread study and have directed the development of various analytical methods for medical applications [17,47]. Platinum nanomaterial-based signal amplification has excellent potential to enhance both the sensitivity and selectivity of electrochemical sensors through remarkable achievements in nanotechnology based sensor platforms [20,48,49]. The achievement of the control of nanoparticle dimensions, morphology, and dispersion is significant for enabling selective and enhanced electrocatalytic activity. Nanoporous materials demonstrate fascinating properties and have enormous potential to facilitate important electrochemical NO sensor applications [50].

**Figure 1:**
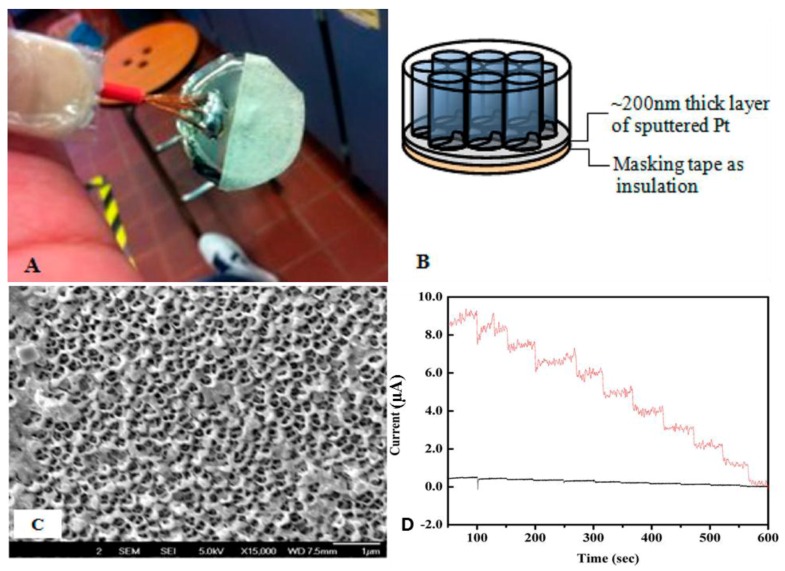
Photograph of the AAO/Pt electrode (**A**) with the general schematic diagram (**B**). FE-SEM of the sputtered Pt (**C**) and (**D**) the amperometric current-time curve response obtained for successive addition of 0.1 μM of NO with Nafion/poly-PtTAPc nanotube/AAO/Pt (red line) and, Nafion/poly-PtTAPc/GCE (black line) for NO concentration of 0.1–1.0 μM in PB (pH 7.4). Adapted with permission from Reference [45]. Copyright American Chemical Society, 2013.

Metallo 4′, 4″, 4‴, 4′′′′ tetra-amine phthalocyanine (MTAPc) complexes were electropolymerized within the pores of a Pt coated anodic nanoporous membrane, to create a high-density nanotube array for amperometric NO sensing [45]. A sputtering technique was employed to coat ~200 nm Pt on the aluminum anodic oxide (AAO) template. Figure 1A displays a photograph of the AAO/Pt electrode, where the masking tape ensures that the MTAPc solution accesses the conductive Pt layer exclusively through the pores of the AAO membrane, as shown in the field emission scanning electron microscopic (FE-SEM) image (Figure 1B). The annular morphology of the AAO/Pt was obtained by the pinching of the tape followed by a brief vacuum treatment (Figure 1C). Platinum nanoparticles entered the nanometric pores and transited to the inside walls of the channel, thus producing an annular morphology. The amperometric curves of the Nafion/poly-PtTAPc nanotube/AAO/Pt (red line) and the Nafion/poly-PtTAPc/GCE (black line) depict the sensing of NO in the range from 0.1 to 1.0 μM (Figure 1D). The design of the nanoporous Pt electrode exhibited sensitivity improvements via the analytes that were adsorbed on its surface as the rate-determining step. The sensitivity of the metal centers (M = Cu^2+^, Zn^2+^, and Pt^2+^) of the poly-MTAPc material were insensitive to the amperometric sensing of NO.

Electrochemical microsensors have been employed for the detection of NO at close proximity to such sources at site-specific NO concentrations. Numerous Pt-based microelectrodes, ultra-microelectrodes, or arrays of microelectrodes have been employed for the detection of NO of interest in biological tissues or cell lines, due to the high rate of mass transfer, rapid electrochemical kinetics, diminutive capacitance, fast charging current decay, excellent signal/noise ratios, and overall reduced dimensions of the electrochemical setup [31,44,51,52,53]. Several investigations have already demonstrated the sensing of NO generated from single neurons in pond snails, and human umbilical vein endothelial cells employing microsensors [54,55]. However, these studies were limited to in vitro NO analysis at a single site.

**Figure 2:**
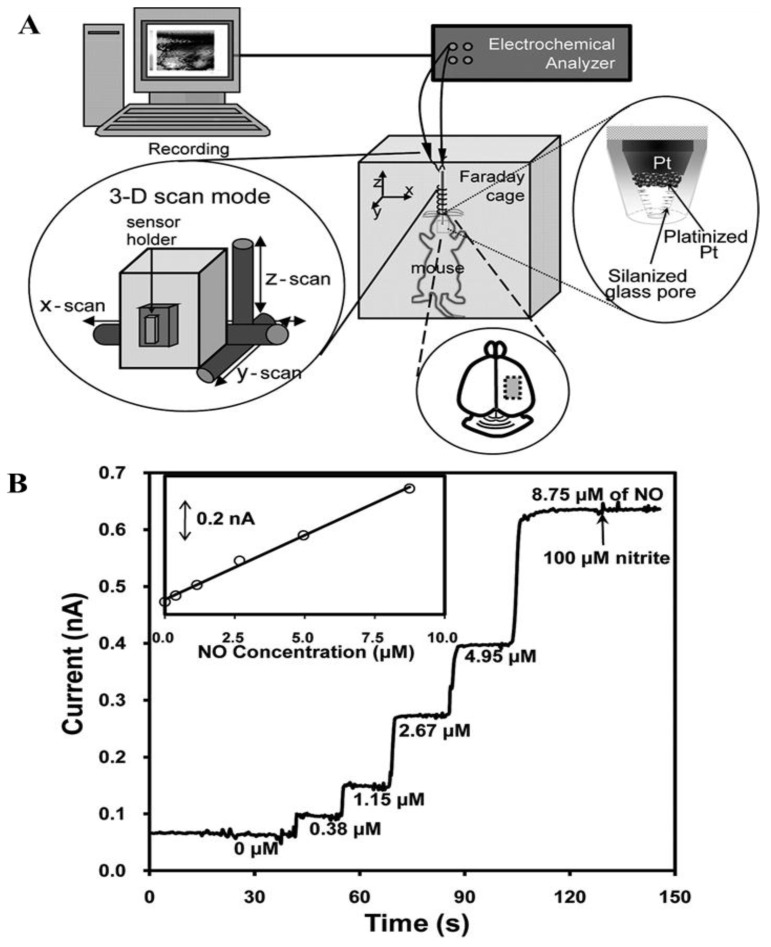
(**A**) Schematic diagram of the experimental setup. SECM is set for a two-dimensional scan at a constant height mode; (**B**) a typical sensor current response curve as a function of the NO concentrations (Inset: corresponding calibration curve). Adapted with permission from reference [57]. Copyright American Chemical Society, 2011.

Furthermore, nanopore-based amperometric sensors coupled with scanning electrochemical microscopy (SECM) have been employed for the two-dimensional imaging of localized concentrations of NO from a NO emitting microdisk polymer film [56]. Lee and co-workers developed an amperometric NO sensor that contained a nanoporous Pt electrode that facilitated the direct electrochemical in vivo imaging of localized NO concentrations at the cortical surface of a living mouse brain [57]. This amperometric sensor was fabricated based on a nanopore electrode (radii < 500 nm) at a Pt-based substrate, with the experimental setup and the sensor response to the addition of NO depicted in Figure 2. The nanopore Pt electrode was fabricated by etching Pt from the Pt nanodisk and the resulting electrode was platinized via electrodeposition using cyclic voltammetry (CV) in a 3% chloroplatinic acid (*v*/*v* in water). The low detection limit (<10 nM), rapid response, and high selectivity for the in vivo imaging of localized NO concentrations under biological conditions were attained via a successful silanization treatment, using a planar-like geometry sensor with a small pore size and extensive active electrode surface area.

## 3. Platinum Nanoparticles

The design of Pt nanoparticle-based electrode materials has a key role in the high performance of electrochemical NO sensing platforms through electroanalytical principles, and has excellent potential for improving both their sensitivity and selectivity. Platinum nanoparticle-based nanocomposites and functional nanomaterials are being designed for the sensing of NO in order to further improve or accelerate catalytic activity, conductivity, and electrochemical signal transduction [19,31]. Nanometric structures comprised of Pt or other metal catalysts may increase reactivity with various analyte molecules through increases in step density as the size decreases and strain is induced via interactions with the support [17,20,34]. Marashdeh and co-workers have investigated the structural, energetic, electronic, and magnetic properties of Pt clusters (Pt*_n_*, *n* = 2–6, 8, 10, 13, 30, and 39) with NO molecules based on the density functional theory (DFT) calculation [58]. It has been shown that smaller dimensioned Pt configurations tend to arrange as planar structures, whereas larger sized clusters are inclined to form compact structures. The Pt clusters (*n* = 2–4) provided the most stable top adsorption sites, and the Pt clusters with *n* = 5, 6, 39 were favorable on bridge sites, suggesting that NO bonds soften more for larger clusters in contrast to smaller clusters.

A nanocomposite comprising Pt nanoparticles (~20 nm) dispersed on a multi-walled carbon nanotube (MWNT)-modified GC electrode (Pt/MWCNT/GC) was fabricated through electrodeposition for NO sensing by Zhang et al. [59]. The detection of NO at the Pt/MWCNT modified electrode was performed using an amperometric method based on the electrocatalytic reduction of NO in 0.1 phosphate buffer (PB, pH 7.0) with a detection limit of 0.1 µM. The Pt/MWCNT/GC electrode displayed high stability toward NO sensing, and only 2% of catalytic activity was lost by performing continuous 100 cycles of CV. Hu and co-workers prepared an electrochemical NO sensor using a GC electrode that was modified with an electrocatalytic film composed of dihexadecyl hydrogen phosphate, Pt nanoparticles, and acetylene black (AB), where Pt nanoparticles were formed by electrodeposition in acidic solution [60]. The developed NO sensor was stable for two weeks and showed good reproducibility. The Pt based nanoparticle modified electrode was successfully applied to the determination of the NO released from rat liver tissue.

**Figure 3:**
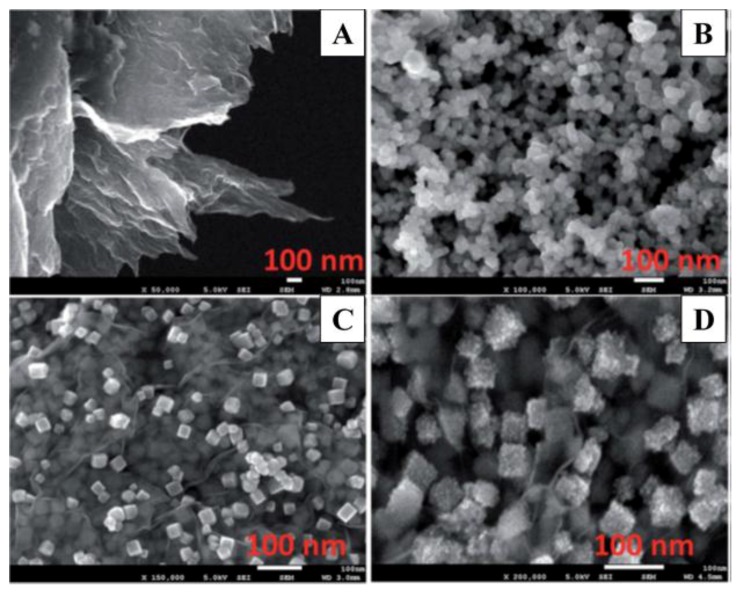
FE-SEM images of the rGO sheets (**A**); Co_3_O_4_ nanocubes (**B**); rGO-Co_3_O_4_ (**C**) and rGO-Co_3_O_4_@Pt nanocomposite (**D**). Reprinted with permission from Reference [42]. Copyright Royal Society of Chemistry, 2015.

Recently, Huang and co-workers demonstrated the amperometric sensing of NO based on a rGO-cobalt oxide nanocube@platinum (rGO-Co_3_O_4_@Pt) nanocomposite electrode [42]. The rGO-Co_3_O_4_@Pt nanocomposite with efficient dispersion of Pt nanoparticles on the Co_3_O_4_ in the rGO sheets was synthesized via a hydrothermal treatment at 180 °C for 12 h with Teflon-lined stainless steel autoclave. Figure 3 presents the FE-SEM images of (a) rGO sheets; (b) Co_3_O_4_ nanocubes; (c) rGO-Co_3_O_4_ and (d) rGO-Co_3_O_4_@Pt nanocomposite materials. As shown in the SEM images of Figure 3, sheet-like graphene structures (Figure 3A) and cubical Co_3_O_4_ nanostructures (Figure 3B) were formed. A SEM image (Figure 3C) of the rGO-Co_3_O_4_ materials, shows that Co_3_O_4_ was highly distributed on the rGO sheets. The Pt nanoparticles were compactly decorated with Co_3_O_4_ with no modification of the Co_3_O_4_ cubical structure, as displayed in Figure 3D. The rGO-Co_3_O_4_@Pt nanocomposite electrode exhibited an improved sensing ability toward NO (in situ generated from NO_2_^−^) when compared with the other modified electrodes. At the nanocomposite electrode, the dispersed Pt nanoparticles with a highly active surface area were in good electrical communication with rGO-Co_3_O_4_ sheets, resulting in an efficient electron transfer process toward the electrochemical oxidation of NO. The developed nanocomposite electrodes exhibited enhanced sensing performance with a low detection limit of 1.7 µM, and showed high stability with good reproducible measurements. The developed rGO-Co_3_O_4_@Pt [42], Pt/MWCNT [59], Pt/AB [60] electrodes exhibited less sensitive than that of the nanopore Pt based electrodes, revealing that the nanopore Pt electrode possessed a high surface area and high catalytic activity toward NO oxidation [45,57].

## 4. Platinum–Gold Nanoparticle/Graphene Nanocomposites

The development of nanostructured bimetallic catalysts has been intensively employed in the area of electrocatalysis and sensors to optimize selective catalytic performance. This was accomplished by controlling the charge transfer between different metals, the local coordination environment, lattice strain, and surface element distribution, particularly toward enhancing their sensitivity and selectivity in contrast to their monometallic analogues, which is often referred to as synergistic effects between the two metals [61,62,63]. The fabrication of metal nanoparticles with graphene enables excellent electrocatalytic properties that lead to high NO sensitivity [23,40,41,64]. Two-dimensional lattice graphene has emerged as a suitable candidate, and attained enormous interest and explosive growth in electrocatalysis and electrochemical sensors. Due to the extraordinary electron transport properties and oxygen functionalities of graphene, it helps to support and accelerate electron transfer kinetics during electrochemical processes [65,66,67]. Based on the first-principles calculations, the adsorption of NO and carbon monoxide (CO) gases on palladium (Pd)- and Pt-decorated single walled carbon nanotubes (SWNT) were studied by Cao and co-workers [41]. It has been reported that Pd and Pt might possibly be adsorbed above the top of axial C–C bond centers. Subsequently, Pd and Pt atoms transit far from the SWNT surface via robust interactions between the gas molecules (CO and NO) and the metal atoms that are present. Duan and co-workers developed flexible electrochemical sensors, based on gold (Au)@Pt core-shell nanoparticle dispersed graphene paper using a chemical reduction strategy with trisodium citrate. The Au@Pt/graphene nanocomposites were closely-packed as a freestanding cell culture substrate for the real-time monitoring of cell secreted NO [23].

**Figure 4:**
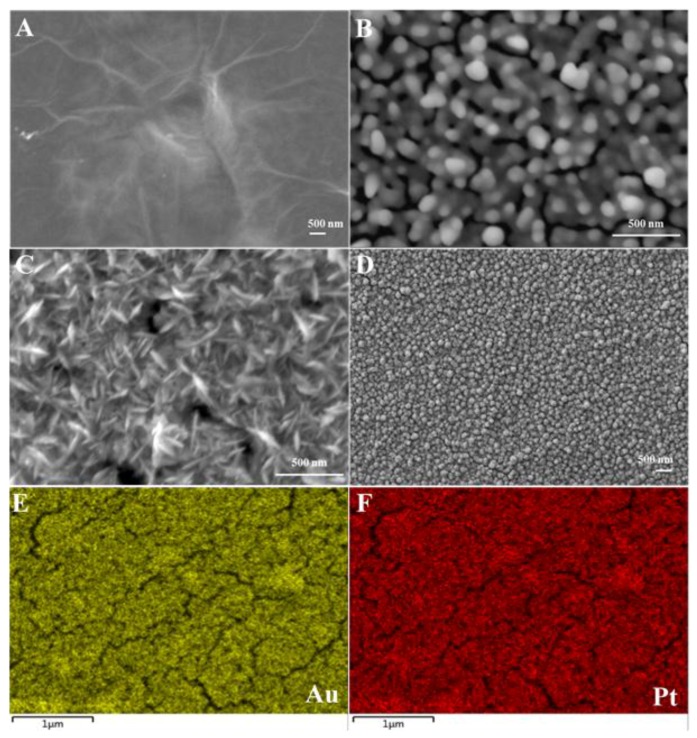
FE-SEM images observed for rGO (**A**) and Au/rGO (**B**); Pt/rGO (**C**) and PtAu/rGO nanocomposites (**D**); the elemental mapping of Au (**E**) and Pt (**F**) of the PtAu/rGO nanocomposites.

**Figure 5:**
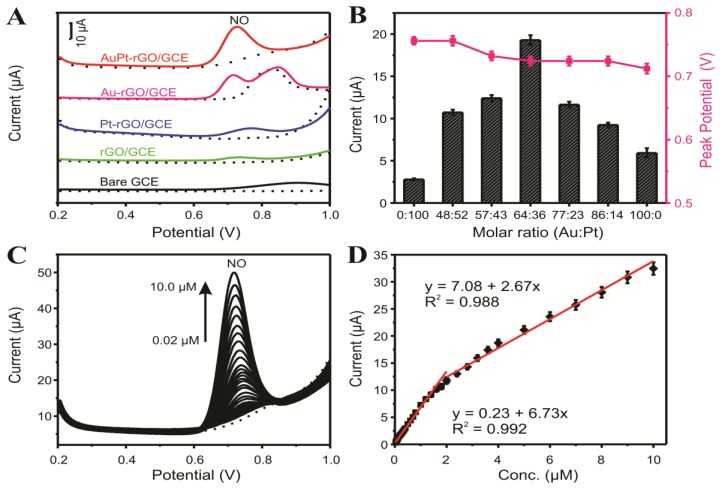
(**A**) DPV responses of 5.0 μM NO on bare GCE, rGO, Au-rGO, Pt-rGO, and PtAu-rGO modified GCE; (**B**) Dependence of peak current and corresponding peak potential of 5.0 μM NO at different Au/Pt molar ratios of the PtAu-rGO modified electrode; (**C**) DPV responses of an PtAu-rGO electrode toward NO with different concentrations; (**D**) Corresponding calibration plot. Electrolyte: 0.1 M PB solution (pH 7.2). Adapted with permission from Reference [40]. Copyright Royal Society of Chemistry, 2016.

Our research team recently devised a facile and environmentally compatible strategy for the fabrication of an electrochemical sensor based on reduced graphene oxide (rGO) and bimetallic PtAu nanoparticles [40]. A two-step electrochemical method was successfully developed to fabricate PtAu/rGO modified GC electrodes. Firstly, rGO/GC electrode was prepared using electrochemical reduction of drop-casted GO (0.3 mg·mL^−1^) film via CV scanning; and secondly, PtAu nanoparticles were electrodeposited on the rGO/GC electrode by applying constant potential of −0.25 V. The FE-SEM images in Figure 4 depict rGO (A) and Au/rGO (B), Pt/rGO (C) and PtAu/rGO nanocomposites (D). The electrode surface was uniformly covered with the electrochemically reduced rGO sheets. Following electrodeposition, dense PtAu nanoparticles were homogeneously formed on the rGO sheets as clearly seen in Figure 4D. An obvious morphological change may be observed for the bimetallic PtAu nanoparticles in contrast to the mono Au and Pt nanoparticles. As displayed in Figure 4E,F, the Au and Pt elements were homogeneously dispersed within the nanocomposite electrode. Figure 5A presents the differential pulse voltammograms (DPV) of a bare glassy carbon (GC) electrode, as well as the GC electrodes modified with rGO, Au-rGO, Pt-rGO, and PtAu-rGO nanomaterials recorded in 0.1 M PB (pH 7.2) in the absence (dotted lines) and presence of 5.0 µM NO (solid lines). The catalytic current response was enhanced at the PtAu-rGO electrode with a well-defined peak for NO oxidation appearing at +0.72 V, in comparison to the Pt-rGO and Au-rGO nanocomposites. The effects of different Au/Pt molar ratios in the PtAu-rGO nanocomposites were further investigated, and their corresponding peak currents and potentials through the variations in the Au/Pt molar ratios are presented in Figure 5B. The optimized molar ratio of Au:Pt was found to be 64:34 based on the electrocatalytic responses to NO oxidation. The attained catalytic performance of the electrodes suggested that the electrochemical oxidation of NO at the Au-rGO electrode was subject to kinetic limitations, and that the formation of the bimetallic PtAu-rGO nanocomposites significantly increased the current response, which led to an efficient synergistic effect due to the modification of the electronic structure of the surface.

Differential pulse voltammetric (DPV) was applied for the detection of NO at optimized PtAu-rGO nanocomposites, as revealed in Figure 5C. The anodic peak currents increased in a linear manner against NO concentrations, which was ascribed to the oxidation of NO. There were two segments in the relevant linear range for the detection of NO, which were 0.02–1.8 μM and 2.0–10 μM, as shown in Figure 5D. The limit of detection (LOD) was calculated to be 3.69 nM (S/N of 3). The obtained limit of detection was much lower than that of other electrodes reported in the literature including rGO-Co_3_O_4_@Pt [42], Pt/MWCNT [59], Pt/AB [60] electrodes. The prepared nanocomposite based sensor exhibited a long-term stability and good reproducibility. Moreover, the developed electrochemical sensor demonstrated a sensitive detection of NO released from rat cardiac cells, revealing that the stressed cells generated considerably more NO than the normal cells.

## 5. Platinum–Tungsten Nanoparticle/Graphene-Ionic Liquid Nanocomposites

Owing to the synergistic electronic effect, PtW bimetallic electrocatalysts may enhance the oxidation of small molecules [68]. It has been established that the chemistry of NO associated with tungsten-based electrocatalysts strongly depends on surface composition and structure. Tungsten atoms have the capacity to release/reduce electron density at their surfaces, which plays a vital role in the adsorption and oxidation of NO. Tungsten atoms have the capacity to reduce the electron density at their surfaces, which play a vital role in the selective adsorption and oxidation of NO [69]. Owing to their exceptional electronic and catalytic properties, bimetallic PtW nanoparticles are widely employed as an exceptional electrocatalyst for various electrochemical reactions [70,71]. Therefore, investigations into electrocatalysts with synergistic bimetallic mechanisms have garnered great attention regarding the emergence of novel functional materials as supports to enhance catalytic performance, as well as the development of NO sensors. Our research group has recently developed an electrochemical sensor based on a platinum–tungsten nanoparticle/graphene-ionic liquid (PtW/rGO-IL) nanocomposite electrode for use with human serum and urine samples [72]. Pt based nanocomposite electrode was fabricated using a single step electrochemical reduction approach. The Pt and W precursors with GO-IL film modified GC electrode was cycled in the potential range between 0.0 and −1.2 V for five cycles at 10 mV·s^−1^.

**Figure 6:**
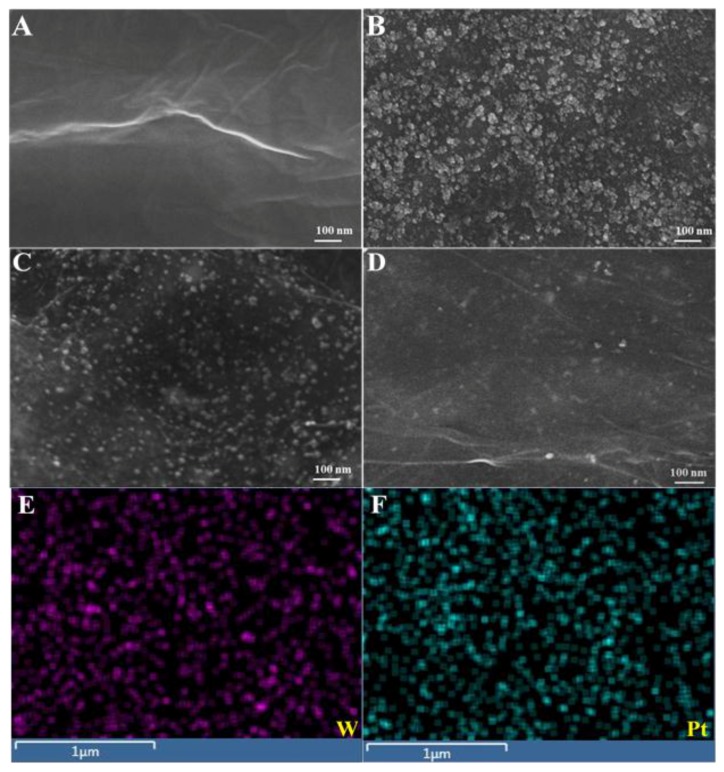
FE-SEM images observed for rGO-IL (**A**) and W/rGO-IL (**B**); Pt/rGO-IL (**C**) and PtW/rGO-IL nanocomposites (**D**); the elemental mapping of Wu (**E**) and Pt (**F**) of the PtW/rGO-IL nanocomposites.

**Figure 7:**
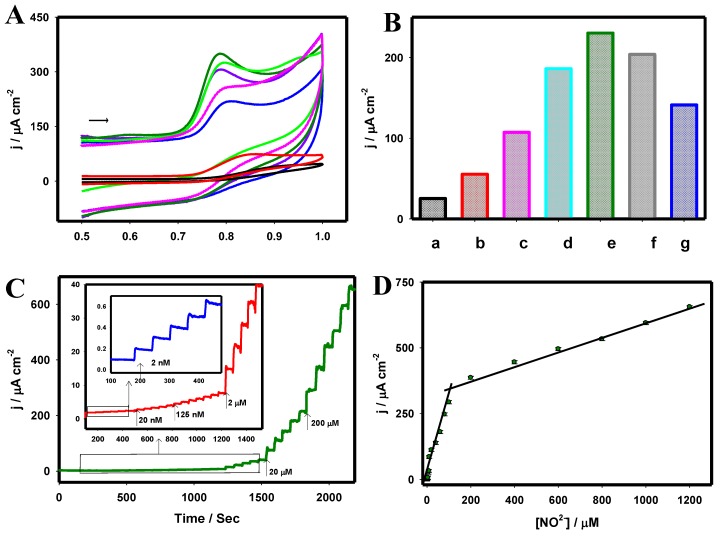
(**A**) CVs of the bare GC (black), rGO-IL (red), W/rGO-IL (blue), PtW(25:75)/rGO-IL (light green), PtW(50:50)/rGO-IL (dark green) and PtW(75:25)/rGO-IL (purple) and Pt/rGO-IL (pink) electrodes recorded for 500 µM NO_2_^−^; (**B**) The correlation plots of j_pa_ against various electrodes; (**C**) Amperometric i-t curve response obtained for the PtW/rGO-IL nanocomposite electrode under various NO_2_^−^ concentrations, *E*_app_: 0.78 V; (**D**) Corresponding calibration plot. Electrolyte: 0.1 M PB (pH: 2.5). Adapted with permission from Reference [72]. Copyright Springer, 2016.

Figure 6 depicts a typical FE-SEM image of the rGO-IL (A), W/rGO-IL (B), Pt/rGO-IL (C), and PtW/rGO-IL at the electrode surface. The formed W and Pt nanoparticles were uniformly distributed in the rGO-IL film, where the W and Pt nanoparticles had average dimensions of ~31.3 and ~16.6 nm, respectively. The larger W nanoparticles were composed of a few small W nanoparticles with average dimensions of ~10.6 nm. As displayed in Figure 6D, PtW nanoparticles were homogeneously dispersed on the rGO-IL thin film, and the average PtW nanoparticle size was estimated to be ~7.3 nm. Figure 6E,F illustrate the elemental mapping of W and Pt on the nanocomposite rGO-IL electrode, revealing the high dispersion of W and Pt nanoparticles. Figure 7A presents the CVs of various electrodes, including bare GC (black) and GC modified with rGO-IL (red), W/rGO-IL (blue), PtW(25:75)/rGO-IL (light green), PtW(50:50)/rGO-IL (dark green), PtW(75:25)/rGO-IL (purple), and Pt/rGO-IL (pink) nanocomposites, recorded in 500 µM NO_2_^−^ in 0.1 M PB (pH 2.5). Figure 7B shows the anodic peak current of the oxidation of NO at the various electrodes derived from the CV curves in Figure 7A, respectively. A well-defined NO oxidation peak was obtained at 0.78 V with the enhanced current densities at the PtW/rGO-IL in contrast to the other electrodes under investigation.

An optimized Pt:W ratio was found to be 50:50 for the formation of PtW/rGO-IL nanocomposite for the electrochemical oxidation of NO. The optimized PtW(50:50)/rGO-IL electrode exhibited a ~9 times higher current than the bare GC electrode with a negative peak potential shift of 110 mV. This significant improvement may be attributed to the synergistic effects of the integration of graphene/IL and PtW nanoparticles in the construction of the sensor. The presence of the graphene and IL not only facilitated the uniform formation of PtW bimetallic nanoparticles with smaller dimensions, but also enhanced conductivity and expedited charge transfer.

As presented in Figure 7C, the steady-state current response was attained within 3 s following each addition of NO_2_^−^ at the PtW/rGO-IL electrode in 0.1 M PB (pH 2.5) at the peak potential of 0.78 V vs. Ag/AgCl. The experimental low detection limit of NO was found to be 2 nM. Figure 7D displays two calibration curves with a relatively low concentration range (2 nM–100 µM) and high concentration range (200 µM–1.2 mM). The decrease in sensitivity at the high concentration range may be attributed to the adsorption of species resulting from the NO oxidation at the electrode surface; thus reducing the catalytic response. The limit of detection was estimated to be 0.13 nM based on the signal to noise (S/N) ratio of 3 from amperometric i-t measurements.

**Figure 8:**
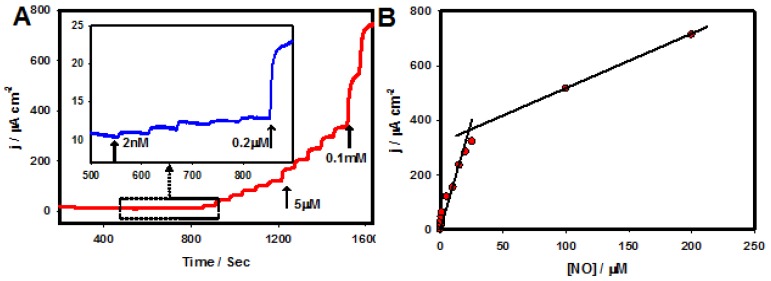
(**A**) Amperometric i-t curve response obtained for the PtW/rGO-IL nanocomposite electrode in 0.1 M PB (pH: 7.4) to various NO concentrations (2 nM to 0.2 mM), *E*_app_: 0.78 V; (**B**) The corresponding calibration plot of current density against NO concentrations.

**Figure 9:**
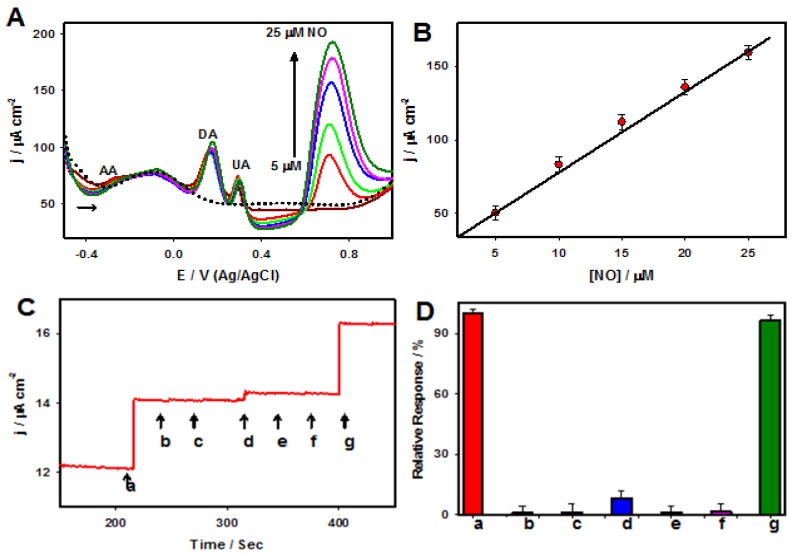
(**A**) DPVs of the PtW/rGO-IL nanocomposite electrode recorded (black, dotted line), 200 µM of DA, AA, and UA (dark red, solid line), 200 µM of DA, AA, and UA with 5 (red, solid line), 10 (green, solid line), 15 (blue, solid line), 20 (pink, solid line) and 25 µM NO (dark green, solid line); (**B**) The corresponding calibration plot; (**C**) Amperometric i-t curve response of the PtW/rGO-IL nanocomposite electrode recorded for 50 nM NO (a), 5 µM NO_2_^−^ (b), 5 µM NH_3_ (c), 5 µM CO (d), 5 µM CO_2_ (e), 5 µM H_2_S (f) and 50 nM NO (g) *E*_app_: 0.78 V; (**D**) The plot of comparison of the NO sensor response to NO in the absence (a) and in the presence (g) of various potential interferents: NO_2_^−^ (b), NH_3_ (c), CO (d), CO_2_ (e) and H_2_S (f). Electrolyte: in 0.1 M PB (pH: 7.4).

Further, the amperometric response to NO was measured in 0.1 M PB (at the physiological pH 7.4) at a constant potential of 0.78 V with NO concentrations that varied from 2 nM to 0.2 mM, as shown in Figure 8A, revealing that the experimental detection limit was 2 nM. The developed electrochemical sensor exhibited high sensitivity over a wide linear range, with a low detection limit toward NO. Furthermore, the interfering effects of electroactive biological compounds such as dopamine (DA), ascorbic acid (AA), and uric acid (UA) were tested for NO sensing based on the DPV technique (Figure 9A,B). As presented in Figure 9A, the peak potentials of the electrochemical oxidation of AA, DA, and UA appeared at −0.28, 0.17, and 0.30, respectively, which were well separated from the peak potential of the electrochemical oxidation of NO (0.76 V). A linear response to NO (0.0 to 25.0 µM) can be observed in Figure 9B, which suggested that the presence of AA, DA, and UA did not interfere with the electrochemical detection of NO.

Moreover, an amperometric i-t technique was employed to test the selectivity of other potential interfering gas species, including of CO, CO_2_, NH_3_, and H_2_S against NO in 0.1 M PB (pH 7.4) (Figure 9C,D). The PtW/rGO-IL nanocomposites based electrode exhibited negligible responses to all of these species, except for CO, which had a slight response (~5%) due to the electrochemical oxidation of CO. The PtW/rGO-IL electrode exhibited high stability and acceptable reproducibility for NO sensing. The PtW nanoparticle/rGO-IL sensor possessed a low calculated detection limit, high sensitivity, a wide linear range, and high selectivity against electroactive interference, which often exists in biological systems, enabling a real-time quantitative sensing capability for NO in human serum and urine samples.

## 6. Conclusions

Significant efforts have been invested in the fabrication of Pt-based nanomaterials as electrode materials with better controlled dimensions, shapes, morphologies, and structures through a large number of solution-phase, electrochemical, and hydrothermal methods, which have demonstrated tremendous promise for electrochemical NO sensing applications. Owing to distinctive physicochemical properties, Pt nanomaterial-based electrochemical NO sensors offer a low detection limit, rapid response, high sensitivity, and selectivity over other active interferents, and have been successfully applied for the detection of NO from living cells, spanning medical diagnostic applications, drug discovery, and biological research. With the objective of encouraging a broader range of importance in their design and fabrication, numerous advances have been realized to facilitate their ultimate use in medicine. Table 1 depicts a summary of the Pt nanomaterials based electrochemical NO sensor platforms that were surveyed in the present review. Recent advances in the construction of Pt nanomaterial-based electrochemical NO sensor platforms and further development of sensing devices at the micro- and nano-scale would provide an auspicious future for the realization of sensitive and selective analytical techniques for biological study and biomedical applications.

## Figures and Tables

**Table 1:** Recent electrochemical NO sensors based on Pt nanomaterials.

Electrode Material	Techniques	Detection Limit	Linear Range	Reference
Nanoporous Pt	Amperometry	32 nM	0.20–1.80 µM	[56]
Nanoporous Pt	Amperometry	10 nM	0.0–8.75 µM	[57]
Nanoporous Pt	Amperometry	10 nM	0.10–1.0 μM	[45]
/MTAPc				
PtO	Amperometry	1 nM	0.50–4.0 μM	[73]
Pt/AAO	DPV	10 nM	0.0–3.40 µM	[44]
Pt	Amperometry	10 nM	-	[74]
Pt	Amperometry	0.50 μM	20–100 μM	[75]
Pt/MWNT	Amperometry	0.10 µM	0.40 µM–0.10 mM	[59]
Pt/AB	Amperometry	50 nM	0.18–120.0 μM	[60]
Pt/Co_3_O_4_-rGO	Amperometry	1.73 µM	10–650 μM	[42]
Pt-Fe(III)	DPV	18 nM	84 nM–0.78 mM	[46]
PtAu/rGO	Amperometry	3.69 nM	0.02–50 μM	[40]
	DPV	2.88 nM	0.02–10 μM	
PtW/rGO-IL	Amperometry	0.13 nM	2 nM–1.20 mM	[72]
	DPV	42.49 nM	0.50 µM–1.0 mM	

MTAPc: metallo 4′, 4″, 4‴, 4⁗ tetra-amine phthalocyanine; AAO: aluminum anodic oxide film; DPV: differen tial pulse voltammetry; MWNT: multi-walled carbon nanotubes; AB: acetylene black; rGO: reduced graphene oxide; IL: ionic liquid.
